# Predicting Cognitive Load and Operational Performance in a Simulated Marksmanship Task

**DOI:** 10.3389/fnhum.2020.00222

**Published:** 2020-07-03

**Authors:** Hrishikesh M. Rao, Christopher J. Smalt, Aaron Rodriguez, Hannah M. Wright, Daryush D. Mehta, Laura J. Brattain, Harvey M. Edwards, Adam Lammert, Kristin J. Heaton, Thomas F. Quatieri

**Affiliations:** ^1^Human Health and Performance Systems Group, MIT Lincoln Laboratory, Lexington, MA, United States; ^2^Department of Biomedical Engineering, Worcester Polytechnic Institute, Worcester, MA, United States; ^3^Military Performance Division, U.S. Army Research Institute of Environmental Medicine, Natick, MA, United States

**Keywords:** cognitive load, predicting performance, multimodal physiological features, virtual environment, marksmanship

## Abstract

Modern operational environments can place significant demands on a service member's cognitive resources, increasing the risk of errors or mishaps due to overburden. The ability to monitor cognitive burden and associated performance within operational environments is critical to improving mission readiness. As a key step toward a field-ready system, we developed a simulated marksmanship scenario with an embedded working memory task in an immersive virtual reality environment. As participants performed the marksmanship task, they were instructed to remember numbered targets and recall the sequence of those targets at the end of the trial. Low and high cognitive load conditions were defined as the recall of three- and six-digit strings, respectively. Physiological and behavioral signals recorded included speech, heart rate, breathing rate, and body movement. These features were input into a random forest classifier that significantly discriminated between the low- and high-cognitive load conditions (AUC = 0.94). Behavioral features of gait were the most informative, followed by features of speech. We also showed the capability to predict performance on the digit recall (AUC = 0.71) and marksmanship (AUC = 0.58) tasks. The experimental framework can be leveraged in future studies to quantify the interaction of other types of stressors and their impact on operational cognitive and physical performance.

## 1. Introduction

Cognitive load is a construct that represents the amount of processing resources (to include working memory) required by a given task (Paas et al., 2003). Individuals systematically vary in both their processing capacity and performance on a complex task. Currently, there is a significant gap in capability to unobtrusively monitor cognitive load in order to mitigate the deleterious effects of fatigue, facilitate learning, and optimize task performance. This is particularly true in modern operational environments, which place significant demands on a service member's cognitive resources due to high physical and mental demands, sleep restriction, and extreme environmental conditions (Friedl, 2012; Choi et al., 2014; Proctor et al., 2017; Smith et al., 2019).

Monitoring cognitive load and task-relevant performance has been demonstrated in the laboratory using modalities such as heart rate (McDuff et al., 2014), skin conductance, pupil dilation (Granholm et al., 1996), neural activity (e.g., electroencephalography Quatieri et al., 2016), gait (Lin and Lin, 2016), speech (Lively et al., 1993; Quatieri et al., 2017), and facial movements (Quatieri et al., 2017). One aspect of cognitive capacity that is frequently quantified is working memory, a neural system for temporary storage and management of information required to carry out cognitive tasks (Oberauer and Kliegl, 2006). McDuff et al. (2014) found that pupil diameter increased monotonically as the number of digits held in working memory increased up to 9 digits.

Estimating cognitive load in many operational environments is challenging due to factors such as movement artifacts and ambient conditions (e.g., noise). Moreover, cognitive load measurement often relies on subjective reports since task difficulty in realistic conditions is often unknown. Virtual environments can be used as a bridge between laboratory studies of working memory toward the development of field-ready systems (Kizony et al., 2010). Several studies have analyzed cognitive factors of marksmanship and how training and performance of the human might be enhanced (Zielinski et al., 2016; Head et al., 2017; Clements et al., 2018; Rao et al., 2018; Smith et al., 2019). Chung et al. (2005) argued that marksmanship is a complex task that is sensitive to “perceptual-motor, cognitive, affective, equipment, and environmental variables” and should be studied in a way that allows for separation of these components. Other factors involved in accuracy include anxiety and prior mental fatigue level (Head et al., 2017). With regard to physiological measurements, Hatfield et al. (1987) epoched electrocardiography and electroencephalography data, finding increased heart rate and occipital energy related to increased arousal.

In the current study we developed a simulated marksmanship scenario with an embedded working memory task that includes movement, gait, and audio-visual stimulation. As participants performed the marksmanship task, they were instructed to remember numbered targets and recall the sequence of digits at the end of the trial. One of the features of this study is that we were able to carefully control the working memory load through the digit span task, while also tracking a variety of performance attributes relevant to marksmanship, such as reaction time, accuracy and error. Our goal in this study is to utilize physiological and behavioral measurements to make predictions of the cognitive workload and task performance.

## 2. Methods

Eight healthy individuals participated in a virtual reality marksmanship study that included a working memory protocol. Participants were instructed to shoot at moving targets while holding in memory single-digit numbers displayed over each target. After the trial, participants recalled the numbered targets, in order of appearance, using a standardized response format. Low and high cognitive load conditions were defined as trials in which three- or six-digits were presented. Physiological and behavioral measures, collected during each trial, were used to predict both the level of cognitive load experienced by the participant, as well as performance on the digit recall task.

### 2.1. Study Participants

Eight healthy participants (7 male) were involved in the study. The mean age was 28.1±7.2 (SD), ranging from 19 to 37 years. Five out of the eight participants had prior experience with rifles. Participants were recruited without regard for sex, as the hypotheses were not dependent on male/female differences. All participants provided written, informed consent prior to participation. Study procedures were approved by the Committee on the Use of Humans as Experimental Subjects, which acts as the Institutional Review Board for the Massachusetts Institute of Technology, as well as the Air Force Human Research Protection Office.

### 2.2. Immersive Virtual Environment

**Figure 1:**
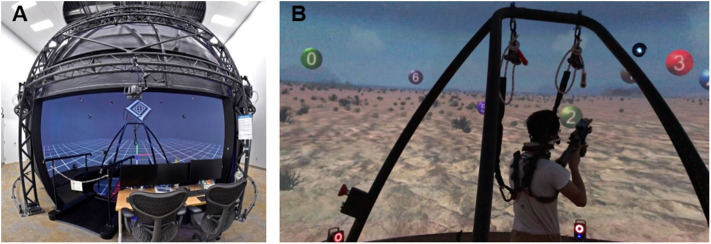
Dismounted marksmanship in the immersive virtual environment. **(A)** Wide-angle view of the MIT Lincoln Laboratory Computer-Assisted Rehabilitation Environment (CAREN) system. **(B)** Participant aiming at a red target during a trial.

We developed an immersive virtual environment for dismounted marksmanship. The visual scene was projected as a seamless spherical image inside the 24-foot diameter Computer-Assisted Rehabilitation Environment (CAREN) system dome (Motekforce Link BV, The Netherlands). Participants were placed in a desert scene (Figure 1) in which they engaged targets using a simulated M4 rifle with realistic shape, material, and weight. The scene and targets were developed and rendered using the Unity video game engine. Targets appeared with uniform distribution in a semicircular shell in front of the participant ranging in azimuth (−90° to 90°) and elevation (−20° to 45°) and moved in linear trajectories. The velocity and elevation of each target were fixed, but sampled from a uniform distribution.

### 2.3. Experimental Protocol

**Figure 2:**
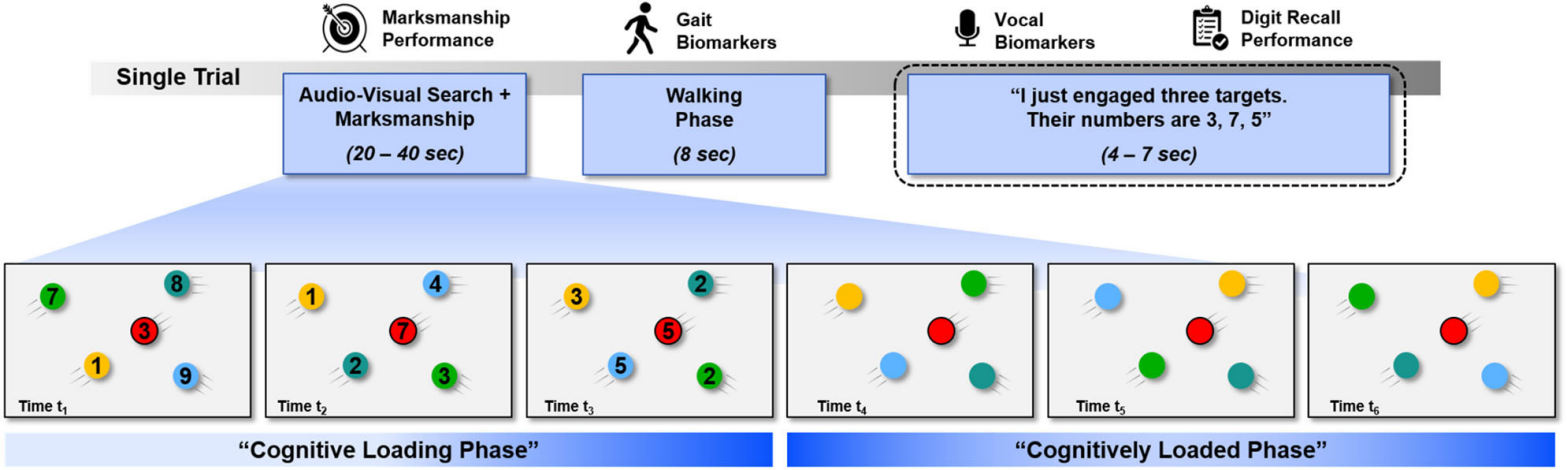
A single trial consisted of three phases: simulated marksmanship, walking, and digit recall. Participants were instructed to acquire just the red targets while holding in memory the digits displayed on the targets. During the digit recall phase, participants recalled the digit sequence held in memory. Timings for the marksmanship and digit recall phases varied by trial and participant, therefore, approximate windows are listed. The walking phase was always the same duration.

The study protocol consisted of six blocks, each of which included 24 trials. Each trial consisted of three phases, including, in order, simulated marksmanship, walking, and digit recall (Figure 2). Each block took approximately 30 min to complete, for a total experiment duration of 3 hours. Setup (beginning) and debriefing (ending) added another hour to the total experimental time.

#### 2.3.1. Simulated Marksmanship Phase

Participants engaged with multicolored targets and were instructed to acquire only red colored targets as quickly as possible while maintaining a high level of accuracy. The marksmanship task was separated into two phases. In the first phase (Cognitive Loading Phase), every target was numbered with a single digit. Participants were instructed to keep in memory the digits presented on the red targets only (either 6 or 3 digits for high and low load conditions, respectively). After the last digit was presented on a red target, the second phase of the marksmanship task was initiated. In this second phase (Cognitively Loaded Phase), participants again were instructed to shoot red targets only, which in this phase were not numbered (see Figure 2), while holding in memory the digits from the red targets they had acquired during the first phase of the task. Presentation of red targets in both phases of the marksmanship task was continuous, with each target appearing on screen immediately after the preceding target disappeared from view.

#### 2.3.2. Walking Phase

The walking phase began as soon as the last red target was acquired. In every trial, Participants were forced to walk at fixed pace on treadmill-like belts for eight seconds and then returned to a halt. In this phase, the load level is also considered constant because all digits are still being maintained in memory.

#### 2.3.3. Digit Recall Phase

In this phase, participants reported the number of targets acquired and recalled the digits presented on these targets. On each trial, participants stated the phrase “Alpha one, this is bravo one. I just engaged N targets,” where “N” is the number of red targets they believed they acquired. Then, they recall the digits of the red targets, e.g., “Their numbers are 3, 7, 5.” The acoustic speech signal during this phase was recorded using a high-fidelity, close-talk directional microphone.

#### 2.3.4. Varying Cognitive Load

The key perturbation in this study was the inclusion of two levels of cognitive load. During the first part of the marksmanship task, either three (low load) or six (high load) numbered targets are presented. In the second part of the marksmanship task, three blank red targets were presented.

Three other experimental parameters were also varied in addition to the level of cognitive load. First, auditory load was varied on a single trial basis by introducing loud, background noise on half the trials. The noise consisted of explosive and impulsive sounds as well as uninformative speech. In the other half the trials, there was no background noise. Second, visual load was varied by altering the number of non-red targets on the visual field. On half the trials, on a single trial basis, there were 20 targets visible (High Load) and in others, there were only 9 targets visible (Low Load). Third, the metabolic load was varied on a block level by running the treadmill at 1.6 m/s (High Load) or 0.8 m/s (Low Load) during the walking phase of the trial. The speed of the treadmill was randomized across blocks (fixed for 24 trials at a time), while other experimental parameters were varied on a trial-by-trial basis (within a block). The order of experimental blocks was randomized across participants.

#### 2.3.5. Phase Duration

The marksmanship phase lasted, on average across participants, 23.2 ± 4.5 seconds for trials with three numbered red targets and 34.8 ± 5.4 s for trials with six numbered red targets. Note that every trial also had three unnumbered red targets (c.f., Figure 2), which is factored into the previously stated time averages. The walking phase was 8 seconds for every trial, regardless of the treadmill pace. The average duration of speech, across participants, was 4.2 ± 1.4 s for recalling three digits and 5.6 ± 1.9 s for recalling six digits. While the duration of trials in this study seem to be longer than typically thought of as working memory, participants reported utilizing a “rehearsal,” process, which is known to extend the duration of working memory through repetition (Atkinson and Shiffrin, 1971; Baddeley, 1992).

### 2.4. Data Acquisition

Behavioral and physiological data recorded included—

- Speech: Recorded using a Sennheiser ME 3 II Dynamic Microphone, which is a light-weight headband microphone (Sennheiser Electronic GmbH & Co., Wennebostel, Wedemark, Germany)
- Heart & Breathing Rate: Measured using the Zephyr BioHarness (Zephyr Performance Systems, Annapolis, MD, USA)
- Body Movement & Gait: Computed from 3D motion using reflective markers placed on anatomical landmarks on participants' legs (Vicon Motion Systems Ltd., U.K.)
- Rifle Movement: Computed from reflective motion capture markers placed on the rifle (Vicon Motion Systems, Ltd., U.K.)

**Figure 3:**
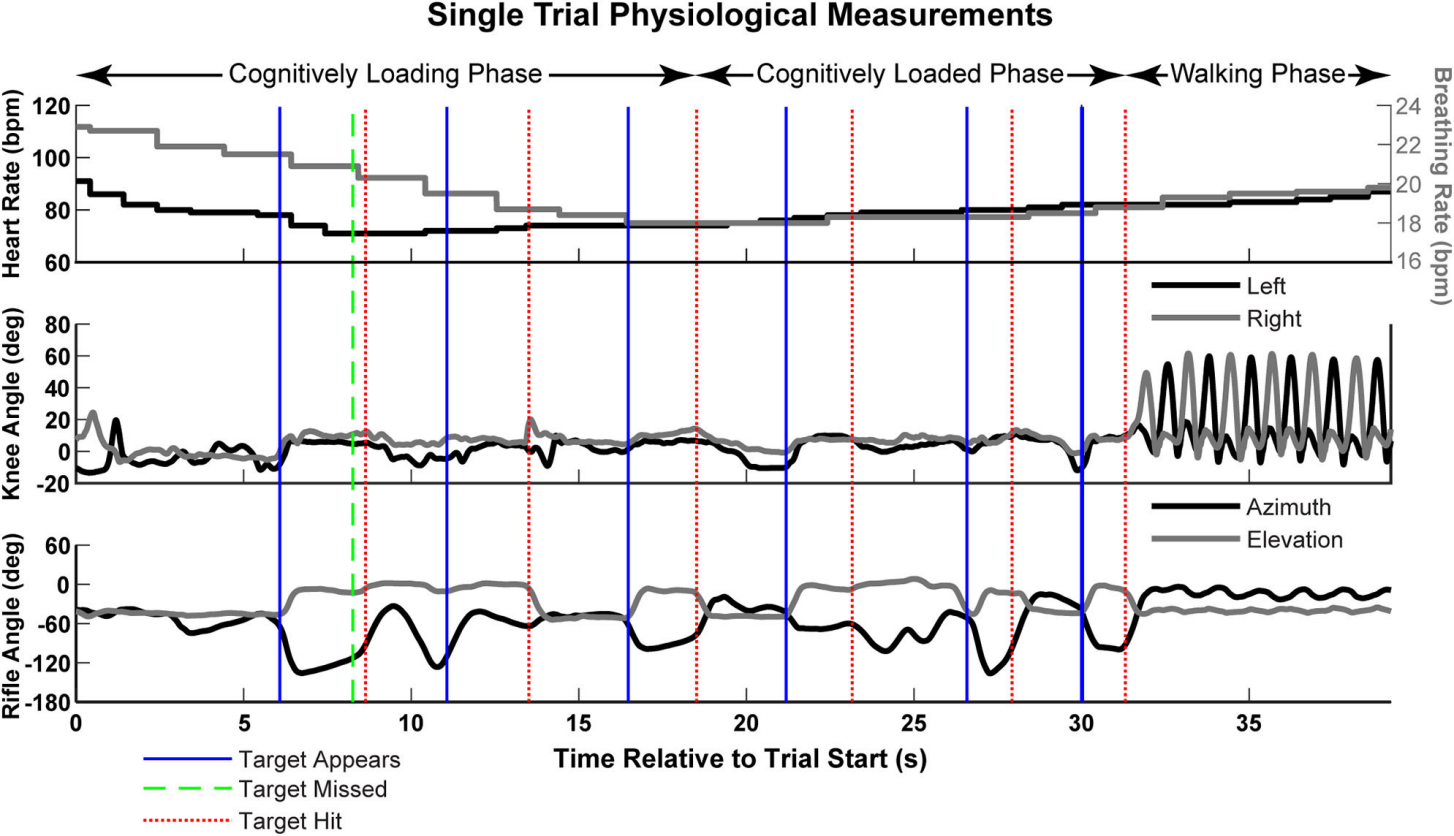
Selected physiological and behavioral measurements from a single trial of the experiment, including heart rate, knee flexion angle, and rifle angle. The solid blue and narrow dashed red lines indicate the appearance and disappearance of each target, respectively. The wide dashed green lines indicate a missed shot. Speech data (verbal digit recall) for the trial is not shown, but would occur immediately after the end of the walking phase.

Selected physiological and behavioral signals from a single trial are illustrated in Figure 3. In this trial, the cognitive load level was low, as denoted by there being three numbered targets (*solid blue lines*), as opposed to six, during the cognitive loading phase. The rifle movement is split into horizontal (*azimuthal*) and vertical (*elevation*) movement. Note that at the appearance of the targets, the participant raises the rifle from a lowered position to an elevated ready position. The rifle is lowered again following acquisition of the targets.

### 2.5. Signal Processing and Feature Calculation

#### 2.5.1. Digit Recall Score

The digits reported by the participant were transcribed by an experimenter *post-hoc* by listening to the audio recordings from the experiment. The degree of accuracy of digit recall was quantified by computing the Levenshtein distance between the correct digit string and the digits the participants reported during the digit recall phase (Navarro, 2001). The digit score was then normalized by the number of numbered red targets presented (i.e., three or six). The smaller the recall score, the smaller the Levenshtein distance and the better the performance. This method robustly factors in insertions, substitutions, or deletions of the digit sequence recalled.

#### 2.5.2. Speech Features

Speech features were computed from the acoustic signal recorded during the digit recall phase and were based on principles of timing and coordination within and across acoustic features (Williamson et al., 2014). For each recording, frame-based articulatory features were first extracted, followed by summary statistics over the recall segment of each trial. A Kalman filter was used to smoothly track the vocal tract resonant frequencies (formants). The dynamics of the first three formants were also included as features (Mehta et al., 2012). Measures of the correlation structure among the vocal tract trajectories were applied to characterize properties of articulatory timing and coordination. Channel-delay correlation and covariance matrices were computed using the three formant trajectories (channels) and contained correlation coefficients between pairwise trajectories of 15 time-delayed versions at three different time delay spacings: 10, 30, and 70 ms. Changes over time in the coupling strengths among the channel signals cause changes in the eigenvalue spectra of the channel-delay matrices. The sum of the eigenvalue log-magnitudes obtained from these matrices quantified the complexity of the signals (Quatieri et al., 2017).

#### 2.5.3. Heart and Breathing Rates

The Zephyr BioHarness sampled an electrocardiogram signal at 250 Hz. Software provided by the manufacturer was used to produce the timing of every detected heart beat (i.e., R-R intervals). That signal was used to compute time-varying heart rate, heart rate variability, root-mean-square of the successive differences, and the proportion of the number of successive R-R interval pairs that differed by more than 50 ms. Additionally, the breathing rate was also computed using the manufacturer's software. These features were computed separately for the two parts of the marksmanship phase and for the walking phase.

#### 2.5.4. Gait, Body, and Rifle

Reflective motion capture markers were placed on the participant's body as well as on the simulated rifle. The forward and backward swing phases of the legs, derived from the movement of the markers, were used to compute gait and gait-related features compensated for treadmill-walking (Zeni et al., 2008). The cadence, measured in steps per minute, was computed as the number of steps taken during the 8 s walking phase. A stride was measured as the window between the toe-off and heal-strike of the same leg (Kharb et al., 2011), and the stride length was the distance the foot traveled between toe-off to heal-strike. Each stride was broken into a swing phase (duration that a leg was off the ground) and a stance phase (duration that the leg was in contact with the ground). For both phases of gait, measures of duration, leg velocity, and joint angles were computed. Force plates, built into the treadmill, were used to determine location and movement of the center of mass. Gait features were only computed during the walking phase of the trial.

Reflective markers on the rifle were used to compute features of rifle movement. These included linear and angular velocities and accelerations as well as integrated path distance during movement. Rifle features were computed separately for the two parts of the marksmanship phase.

### 2.6. Statistical Analyses

Our statistical methods sought to predict both the cognitive load level and the performance on the task in the virtual environment, utilizing the aforementioned physiological and behavioral measurements. Performance-related variables were captured through the software, and included: number of digits recalled, number of misses, reaction time (time between the target appearing and being hit), and the target error (minimal distance between true target location and the vector pointing in the direction of the rifle).

Binary classifiers were used to predict load level (*numberofdigits* = 3) or (*numberofdigits* = 6) as well as to predict other discrete performance variables. Classification was performed in a leave-one-subject-out fashion using version 0.21.2 of Scikit-learn's ensemble.RandomForestClassifier (Pedregosa et al., 2011). The random forest was run with 1,000 estimators and max depth of 4 for each tree. Scikit-learns' RobustScaler was estimated on each fold of the training data and applied to the test participant. Estimates were generated with all features and using a subset of features based on the modality (gait, speech, etc.).

## 3. Results

Physiological and behavioral features, measured non-disruptively, were used to predict the cognitive state of the individual (cognitive load) and an objective metric of performance (digit recall score). These results show that both outcomes can be predicted with a high level of accuracy on a single trial level.

### 3.1. Impact of Load Level on Digit Recall

**Figure 4:**
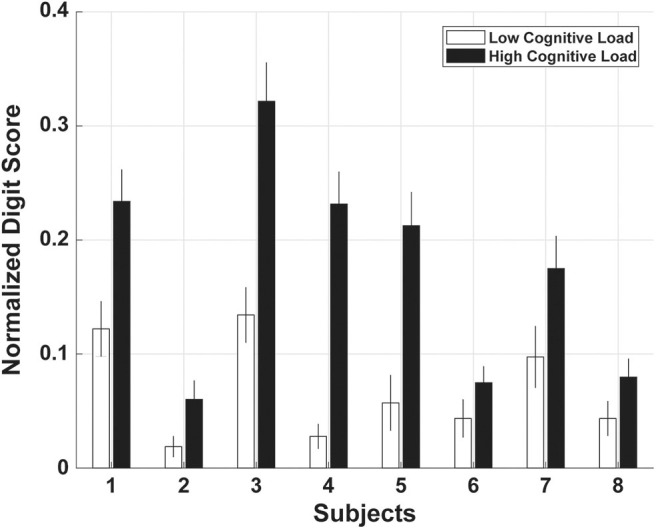
The normalized digit score, based on a Levenshtein distance between the correct digits and the ones reported by the participant, represents the accuracy of the digit sequence recalled. Every participant showed significantly lower performance (higher distance score) in digit recall for the condition with high load level (black bars) compared to the low load level (white bars). Data show average and standard errors of all scores within each of the two conditions.

The greater the level of experimentally induced cognitive load, the less accurate participants were in recalling the digits (Figure 4). All participants had a significantly higher digit score distances (i.e., more inaccuracies) in trials where they had to recall six digits as opposed to three digits [repeated-measures analysis of variance, *F*_(1, 6)_ = 7.69, *p* = 0.032].

### 3.2. Predicting Load Level

**Figure 5:**
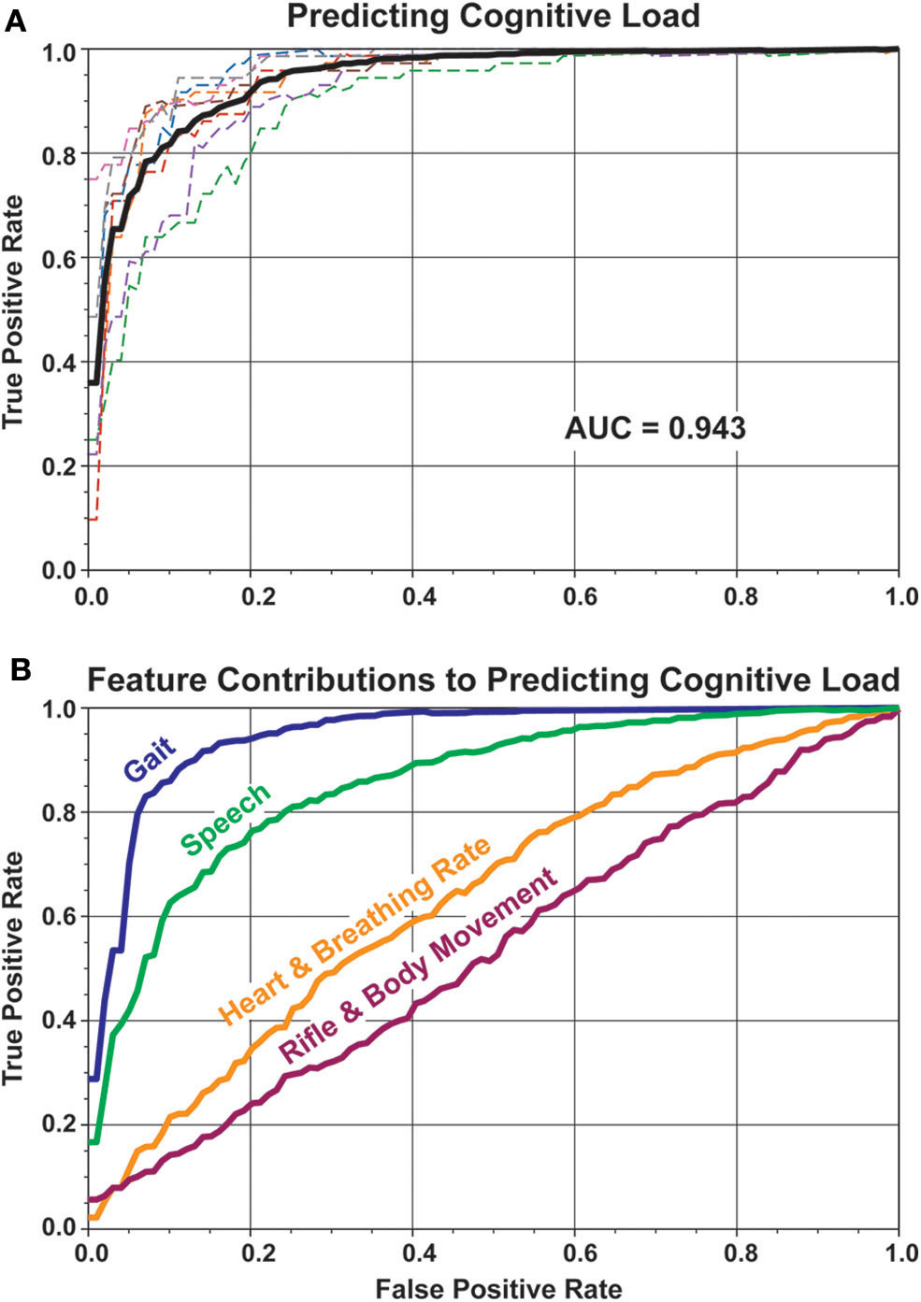
ROC curves show the performance of predicting the cognitive level using physiological and behavioral features. **(A)** All features were used in performing the prediction. Dashed lines show classification performance for each participant, iteratively held-out during training and test. The average area under the ROC curve (AUC) (solid black line) is 0.943. **(B)** Subsets of features were used to predict cognitive load of the trial. Gait features performed best (AUC = 0.950), followed by speech features (AUC = 0.856), then heart and breathing rate related features (AUC = 0.636), and lastly, rifle and body movement features (AUC = 0.534).

A random forest classifier was used to predict the cognitive load level of each trial using all the features combined. Each participant was iteratively held out as the test set during classifier training. Results shown in Figure 5A are only for held out data across the iterations. It is important to consider that these results were gathered on a single trial basis, where at most tens of seconds of data was used in creating a prediction.

### 3.3. Relative Contribution of Features

To delineate the contribution of the categories of features, the classification of single trial cognitive load was performed again, but independently with each of the four feature categories (Figure 5B). The categories were gait, speech, heart, and breathing rate, and movement features. Of these four categories, the gait features predicted the level of load the best (AUC = 0.947) followed by the speech features (AUC = 0.775). The heart/breathing rate features (AUC = 0.564) and body/rifle movement (AUC = 0.507) did not perform above chance.

**Figure 6:**
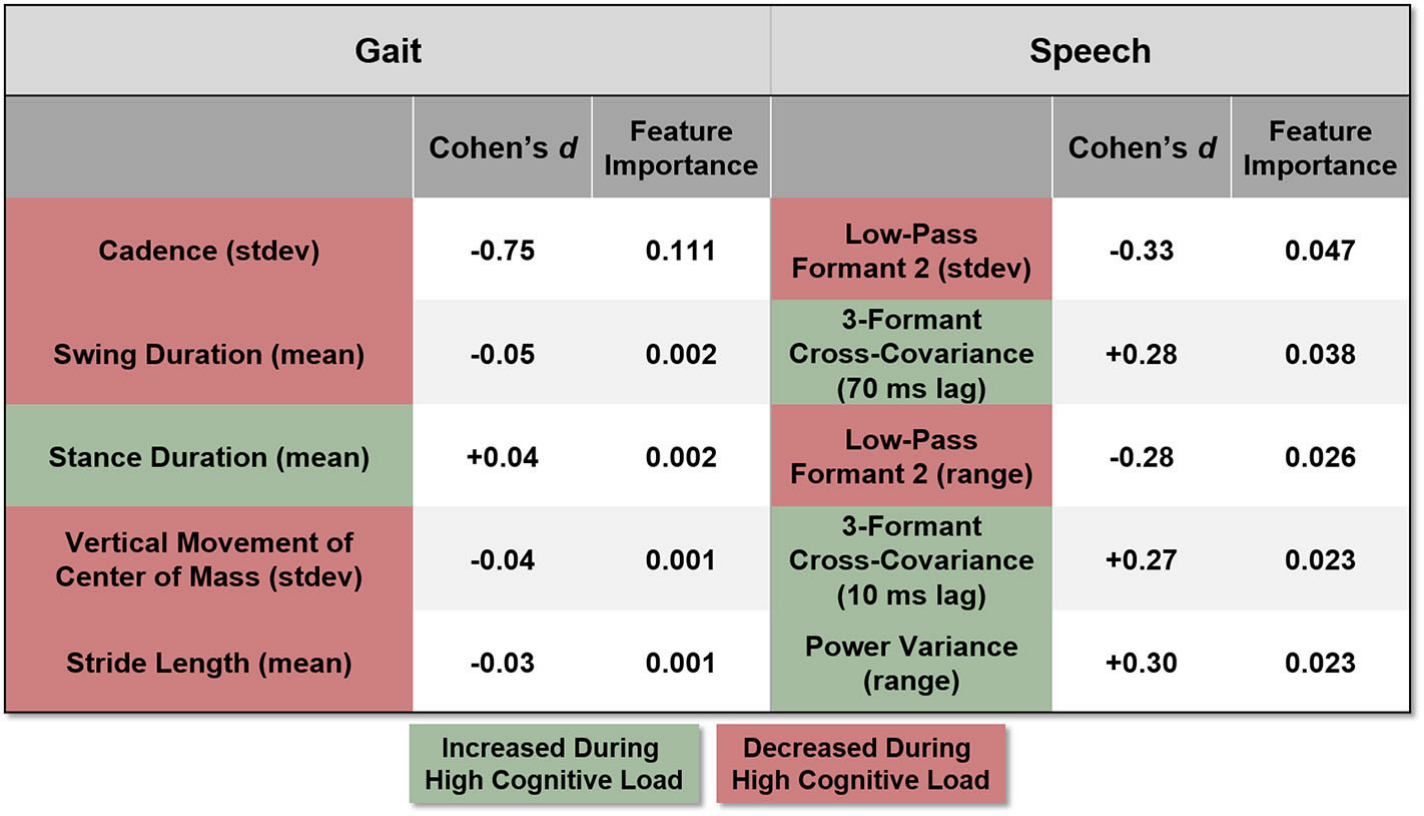
The most discriminative features within each category are listed. Colors indicate whether the feature value increased (green) or decreased (red) during the high cognitive load condition as compared to the low cognitive load condition. The feature importance values, generated from the random forest algorithm, represent a normalized value across all the features. The Cohen's *d* value shows the effect size for that feature in discriminating the load levels.

Within each category of features, the top five most important features are listed in Figure 6. The cells are colored based on the direction of change in the feature between the high and low load conditions. For example, the standard deviation of cadence (colored red), was lower during high load conditions implying slower walking when participants were cognitively loaded. The numbers denote the relative feature importances, across all the features, ranked by the random forest classification approach. The higher the value, the more important the feature was at predicting the load level. For example, the importance of cadence (0.11), was orders of magnitude higher than the next four gait features (0.002–0.001). In the speech feature category, the top five features shared similar levels of importance and by extension, many of the speech features were relatively informative. Recall that the gait features were only computed during the walking phase of the trial (8 seconds of data), and the speech features were computed during the digit recall phase (5–6 s of data). Features within the Heart and Breathing Rate and Rifle and Body Movement categories were excluded from the figure since their respective AUCs (Figure 5B), were much smaller than the Gait and Speech categories.

### 3.4. Predicting Performance

**Figure 7:**
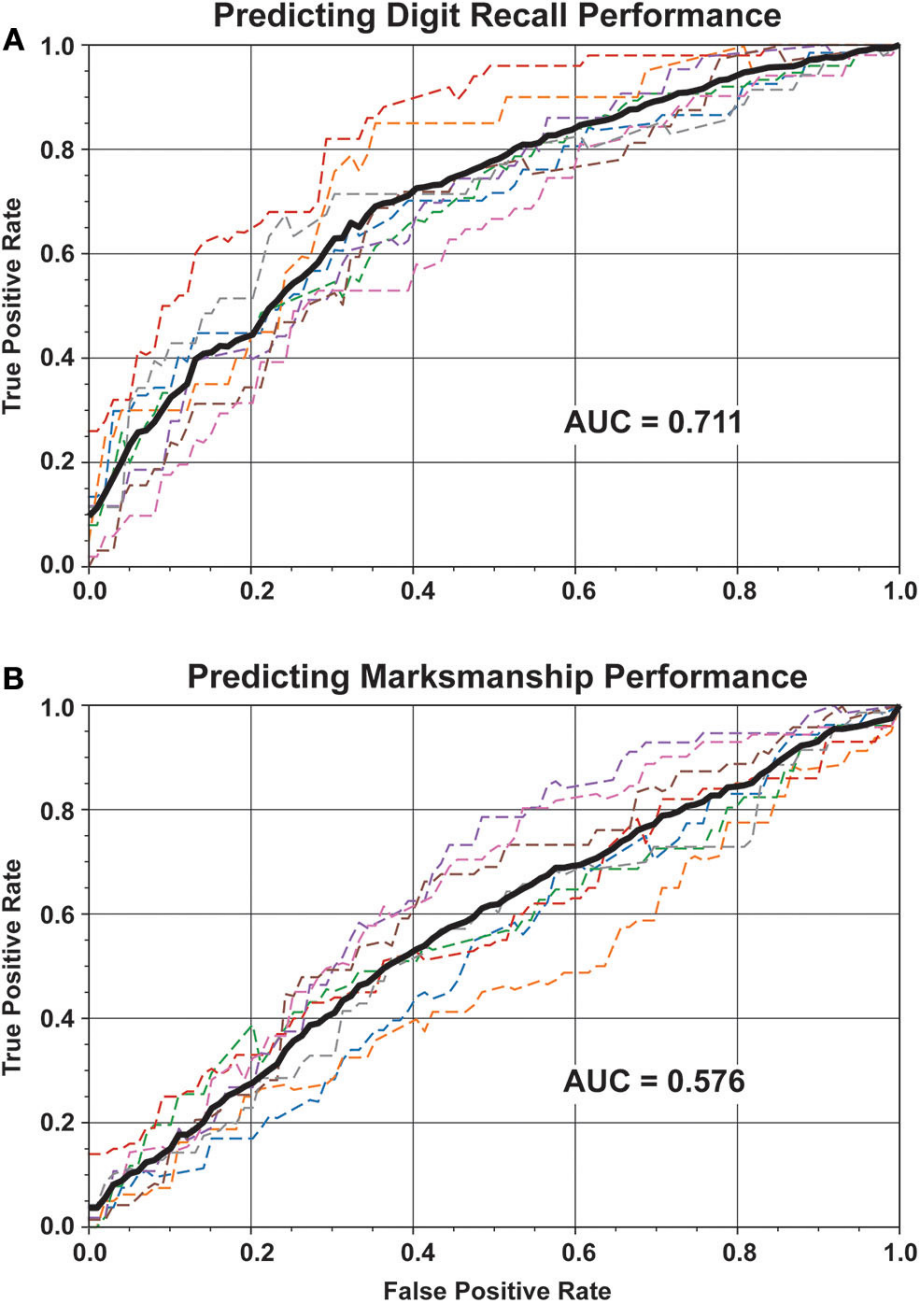
Physiological and behavioral features were used to detect decrements in either digit recall or marksmanship performance. Performance for each participant is shown in dashed line, while the average performance across participants is shown in solid black. **(A)** Performance on the digit recall was classified into whether the recall was perfect or not. Across participants, the AUC was 0.711. **(B)** Focusing specifically on the “Cognitively Loaded” part of the marksmanship phase, classes were defined as either perfect marksmanship acquisition of the red targets or the presence of inaccurate shots to non-red targets. Across participants, the average detection performance was AUC = 0.576.

Predicting performance at the single-trial level is an important initial step toward predicting performance in operational conditions. In addition to estimating the cognitive load of the participant, the digit recall and marksmanship performance was predicted using all the features (Figure 7). Digit recall performance was converted into a binary class problem: either participants were 100% accurate (i.e., digit score = 0) or there was an error (i.e., digit score > 0). Using a random forest classifier, the prediction performance yielded an AUC = 0.711 on average. In similar fashion, marksmanship performance as split into two classes: 100% accuracy in hitting the red targets or less than perfect accuracy. Note that only data within the “Cognitively Loaded” phase of the marksmanship task were used for this analysis. Prediction of marksmanship performance yielded an AUC = 0.576.

## 4. Discussion

We developed a marksmanship scenario with an embedded working memory task in an immersive virtual environment. Using physiological and behavioral features to predict cognitive load yielded a high level of classification accuracy (AUC = 0.943). Of the feature categories measured, gait-related features, followed by speech features, were the most important for accurately predicting performance. The features were also successful at predicting the digit recall performance (AUC = 0.711).

Walking is kinematically structured, showing relatively invariant spatiotemporal correlation (Daffertshofer et al., 2004). However, the cognitive-motor dual task has been shown to impact gait (Lindenberger et al., 2000; Huang and Mercer, 2001). In the present study, participants were required to maintain a string of digits (3 or 6) in memory while walking. Participants showed more stereotypical walking speeds, more time with feet on the ground (decreased swing phase duration and increased stance phase duration), and shorter stride lengths when holding in memory 6-digit strings compared to when they were required to remember 3-digit strings. In aggregate, the behavior points to a slower and more stable gait pattern. In the comparative analysis of feature categories, gait proved to be the most informative. These results build on our previous work, in which we demonstrated that acoustic recordings and video of the face during speech could achieve above chance performance in detecting load level on a laboratory digit span task (Quatieri et al., 2017; Sloboda et al., 2018).

The top-five acoustic speech features reflected changes in the dynamical complexity of the articulatory behavior between the low and high cognitive load conditions. Relative to the low cognitive load condition, the two cross-correlation features indicated that the dynamical complexity of speech increased during the digit recall phase for high-load trials. This result is in line with prior work showing a similar increase in dynamical complexity in a laboratory protocol studying auditory working memory (Quatieri et al., 2015, 2017). Two additional features related to the variation in the second formant indicated that speech during the high cognitive load condition exhibited a more limited formant frequency range. A reduction in the variability of the second formant is analogous to observations in speakers with clinically diagnosed articulatory deficits (e.g., dysarthria) who exhibit reduced second formant variation due to restricted articulatory kinematics (Kim et al., 2009). Taken together, changes in the speech features show the reduction in neuromotor function and flexibility during high cognitive load, just as was seen in the gait behavior.

The accuracy with which speech and gait features can be used to estimate cognitive load suggests that such an approach could provide valuable insight into a service member's cognitive state in operational settings. However, before such a goal can be achieved, it is necessary to move from a laboratory environment into the real world. In the present study, laboratory gold-standards are used to record speech (a high-fidelity acoustic microphone) and gait (3D marker-based motion capture). The acoustic microphone is already suitable for field use, but the marker-based motion capture system is not. In the future, we will explore replacing the motion capture system with accelerometers, which can be placed on footwear or on the legs and have already shown promise in providing gait-related features in field environments (Williamson et al., 2015).

In an operational condition, it would be crucial to predict if an individual were likely to make an error due to cognitive overburden (Friedl, 2018). These results point to the feasibility of using physiological data to predict level of performance on an operationally relevant task. Further, the performance is predicted on a cognitively focused task (digit recall) as well as on a visual-motor-focused task (marksmanship). Signals measured in this study are collected in a non-disruptive way, maintaining the focus on the task, and implying capability to directly apply this approach to other marksmanship and operational paradigms.

Not reported in detail in this paper is the lack of significant effects of noise, treadmill speed, and the number of visual distractors on cognitive performance and marksmanship accuracy. The lack of significant finding might be attributed to separate resources for these modalities (Wickens, 2002), but is likely to be very task dependent (Wang and Duff, 2016). Although we chose a very distracting background noise, the levels were moderate (<85 dB SPL) and were likely not sufficient to force changes in attention due to rapid adaptation to the stimuli. There are mixed results in the literature on the effect noise can have on cognitive performance. The interference, if present, is less likely to impact non-auditory cognitive tasks such as visual reaction time (Molesworth et al., 2015). Since there is no auditory task consistent with the theory of separate resource pools (Wickens, 2002). A future study might incorporate an auditory-based task, such as monitoring a radio or providing auditory cues for the target, which would likely impact performance to a much greater extent as found in a study of driving (Murphy and Greene, 2017). With regard to the number of visual distractors, the most significant findings were a greater amount of time required to hit the target and a higher number of misses, both of which were expected. Not explicitly tests in the current experimental paradigm is the effect that salient colors (e.g., red targets) might have on attention and memory, or if any gender differences exist. All targets were presented in red and the main manipulation was the number of red targets shown. In future studies, the interaction of color and recall can be tested by varying the color of the target within or across trials.

## 5. Conclusions

To rigorously explore the influence of cognitive load on operational tasks, we developed a simulated marksmanship scenario with an embedded working memory component. We demonstrated the capability to discriminate levels of cognitive load and predict performance on an operationally relevant marksmanship task using passively recorded physiological and behavioral signals. In future studies, this experimental framework can be extended to study the interactions of other types of cognitive, perceptual, or physical loads, and their impact on operational performance.

## Data Availability Statement

The raw data supporting the conclusions of this article will be made available by the authors, without undue reservation.

## Ethics Statement

The studies involving human participants were reviewed and approved by MIT Committee on the Use of Humans as Experimental Subjects. The patients/participants provided their written informed consent to participate in this study. Written informed consent was obtained from the individual(s) for the publication of any potentially identifiable images or data included in this article.

## Author Contributions

HR, CS, AL, and TQ designed the experiment and laid out experimental procedures. AR developed the virtual scenario in Unity. AR, HW, and HE performed data collections. HR, CS, DM, HW, and AL performed the data analysis. HR, CS, DM, LB, KH, and TQ drafted the manuscript. All authors contributed to manuscript revision, read and approved the submitted version.

## Conflict of Interest

The authors declare that the research was conducted in the absence of any commercial or financial relationships that could be construed as a potential conflict of interest.
